# Assessment of the Electronic Retinal Implant Alpha AMS in Restoring Vision to Blind Patients with End-Stage Retinitis Pigmentosa

**DOI:** 10.1016/j.ophtha.2017.09.019

**Published:** 2018-03

**Authors:** Thomas L. Edwards, Charles L. Cottriall, Kanmin Xue, Matthew P. Simunovic, James D. Ramsden, Eberhart Zrenner, Robert E. MacLaren

**Affiliations:** 1 Nuffield Laboratory of Ophthalmology, University of Oxford, United Kingdom; 2 Oxford Eye Hospital, Oxford University Hospitals NHS Foundation Trust, Oxford, United Kingdom; 3 Institute for Ophthalmic Research, University of Tübingen, Tübingen, Germany; 4 Werner Reichardt Centre for Integrative Neuroscience, University of Tübingen, Tübingen, Germany

**Keywords:** AFC, alternate forced choice, BaLM, Basic Light and Motion, cpd, cycles per degree, FST, full-field stimulus test, logMAR, logarithm of the minimum angle of resolution, NHS, National Health Service, RP, retinitis pigmentosa

## Abstract

**Purpose:**

To report the initial efficacy results of the Retina Implant Alpha AMS (Retina Implant AG, Reutlingen, Germany) for partial restoration of vision in end-stage retinitis pigmentosa (RP).

**Design:**

Prospective, single-arm, investigator-sponsored interventional clinical trial. Within-participant control comprising residual vision with the retinal implant switched ON versus OFF in the implanted eye.

**Participants:**

The Retina Implant Alpha AMS was implanted into the worse-seeing eye of 6 participants with end-stage RP and no useful perception of light vision. Eligibility criteria included previous normal vision for ≥12 years and no significant ocular or systemic comorbidity.

**Methods:**

Vision assessments were scheduled at 1, 2, 3, 6, 9, and 12 months postimplantation. They comprised tabletop object recognition tasks, a self-assessment mobility questionnaire, and screen-based tests including Basic Light and Motion (BaLM), grating acuity, and greyscale contrast discrimination. A full-field stimulus test (FST) was also performed.

**Main Outcome Measures:**

Improvement in activities of daily living, recognition tasks, and assessments of light perception with the implant ON compared with OFF.

**Results:**

All 6 participants underwent successful implantation. Light perception and temporal resolution with the implant ON were achieved in all participants. Light localization was achieved with the implant ON in all but 1 participant (P4) in whom the chip was not functioning optimally because of a combination of iatrogenic intraoperative implant damage and incorrect implantation. Implant ON correct grating detections (which were at chance level with implant OFF) were recorded in the other 5 participants, ranging from 0.1 to 3.33 cycles/degree on 1 occasion. The ability to locate high-contrast tabletop objects not seen with the implant OFF was partially restored with the implant ON in all but 1 participant (P4). There were 2 incidents of conjunctival erosion and 1 inferotemporal macula-on retinal detachment, which were successfully repaired, and 2 incidents of inadvertent damage to the implant during surgery (P3 and P4).

**Conclusions:**

The Alpha AMS subretinal implant improved visual performance in 5 of 6 participants and has exhibited ongoing function for up to 24 months. Although implantation surgery remains challenging, new developments such as OCT microscope guidance added refinements to the surgical technique.

Inherited retinal degenerations are the most common cause of blindness in the working-age population,^1^ yet a scarcity of treatment options exist for this group of patients. Although the prospect of targeting degenerate photoreceptors with retinal gene therapy holds promise, an alternative strategy for patients with end-stage disease may be required. In this setting, an electronic retinal prosthesis can subsume the role of phototransduction by directly stimulating the remaining inner retina in response to incident light. Only 4 devices have been granted CE Mark for commercial use in the European Economic Area: Retina Implant Alpha IMS (first-generation device, Retina Implant AG, Reutlingen, Germany), Retina Implant Alpha AMS (second-generation device, Retina Implant AG), Argus II Retinal Prosthesis System (Second Sight Medical Products Inc, Sylmar, CA), and IRIS II (Pixium Vision, Paris, France). The key distinction between the Alpha devices and the Argus II or IRIS II systems is that the former consists of a photodiode array located in the subretinal space (i.e., in the anatomic plane of the degenerate photoreceptors), whereas the latter 2 comprise an epiretinal array located on the surface of the retina.^2^ A further difference is that the Alpha implant performs both light detection and charge transfer to the overlying inner retina, whereas the Argus II and IRIS II use a spectacle-mounted digital camera to detect incident light that is then transmitted wirelessly to the implant's receiver.^2^ A small number of key changes were introduced into the latest Alpha device to prolong its functional lifespan. These included (1) switching from a monophasic (direct current) to a biphasic (alternating current) pulse, (2) a new and slightly larger chip comprising 1600 stimulation units (the IMS had 1500), and (3) a necessarily wider polyimide foil to accommodate the new chip. No significant change to the implantation surgery was required.

We set out to assess the safety and efficacy of the Alpha AMS, the latest iteration of the subretinal implant, in patients with profound visual loss from end-stage retinitis pigmentosa (RP). A cohort of 6 participants were enrolled over a 12-month period as part of the Oxford Retinal Implant Alpha AMS Trial sponsored by the University of Oxford and independently funded by an Invention for Innovation (i4i) award from the National Institute for Health Research. The study was registered as a clinical trial (www.clinicaltrials.gov NCT02720640). The views expressed are those of the author(s) and not necessarily those of the National Health Service (NHS), the National Institute for Health Research, or the Department of Health.

## Methods

### Participants and Eligibility Criteria

Six patients with advanced RP, aged 45 to 63 years (mean, 53 years), were recruited to the trial after National Research Ethics Committee approval (ref. 15/LO/0445) via the outpatient clinics of the Oxford Eye Hospital in the Oxford University Hospital's NHS Foundation Trust. Key eligibility criteria were nonuseful light perception or no light perception vision in 1 or both eyes, a period of normal visual function for ≥12 years, no additional ocular pathology, no significant systemic diseases, and a reliable electrically evoked phosphene response in the eye receiving the implant.

Informed consent was obtained, and the research followed the guidelines of the Declaration of Helsinki (seventh revision, 2013). A senior optometrist and an ophthalmologist assessed baseline vision independently. In all 6 participants, this was recorded as vague nonlocalizing perception of light in the eye to be implanted, which was also pseudophakic. Residual inner retinal function was confirmed by eliciting an electrically evoked phosphene response^3^ with active Dawson, Trick, and Litzkow electrodes and an OkuStim device (Okuvision GmbH, Kusterdingen, Germany). The genetic mutation was known for all 6 participants (Table 1), and there was no other significant ocular pathology (e.g., glaucoma). Participants underwent 3 days of vision testing and assessments at months 1 (implant switch on), 2, 3, 6, 9, and 12 (conclusion of study). Ongoing off-study visits continued after 12 months as per standard NHS care at this center.

**Table 1:** Clinical Details

Study Code	Age, yrs	Gender	Affected Gene	Operated Eye	Follow-up (mos)
P1	45	F	*USH2A*	L	24†
P2	49	F	*PDE6B*	R	24†
P3	48	F	*RPE65*	R	3∗
P4	57	F	*USH2A*	R	9
P5	54	M	*RPGR*	L	6
P6	63	F	*CERKL*	L	3

∗ Explanted after conjunctival revision surgery at 3 months follow-up.

† Off-study follow-up past 12 months.

### Alpha AMS Subretinal Implant

The light-detecting subretinal component of the Alpha AMS comprises a 4.0 × 3.2-mm × 70-μm silicone microchip coated on 1 side with 1600 stimulation units. It was designed by the University of Ulm, Institute for Microelectronis^4^ and produced by AMS AG (Premstätten, Austria). Each stimulation unit has a dimension of 70 × 70-μm and includes a photodiode, an amplifier, and a stimulation electrode. Each unit cluster on the chip surface stimulates the overlying retinal tissue with a current according to the local light intensity, with the sensitivity of the chip adjusted by the participant to suit the ambient light intensity. The chip requires a power supply to convert incident light into a current detectible by the adjacent cells of the overlying inner retina. Power reaches the chip via the polyimide foil, which connects through a silicone cable to a sub-periosteal ceramic-housed induction coil that couples with an external inductive patch attached magnetically behind the ear. The participant carries a power supply box (that uses regular household AA batteries), which has controls for adjustment of signal gain and sensitivity in the amplifiers integrated into each of the 1600 stimulation units in response to ambient light conditions. The principal design of this approach was developed by the SUBRET-consortium,^5^ and proof-of-concept was shown in 2 pilot studies.^6^, ^7^

### Implantation Surgery

**Figure 1:**
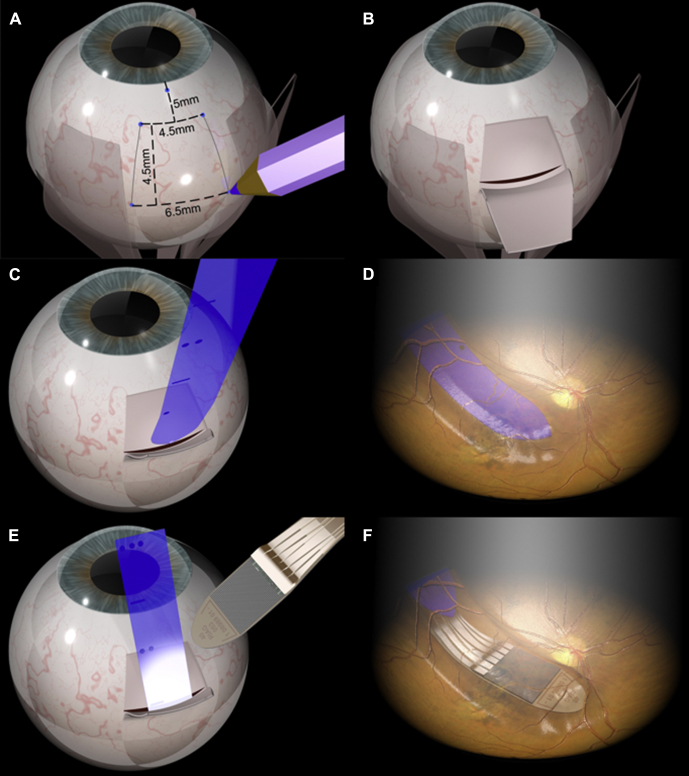
Implanting the subretinal chip. A fornix-based partial thickness scleral flap is constructed (**A**, **B**). After vitrectomy and localized retinal detachment, the guide foil is inserted through a full-thickness slit incision in sclera and choroid (**C**). Once the foil is positioned under the fovea, the chip is advanced along the same path before removal of the guide foil (**D**–**F**).

**Figure 2:**
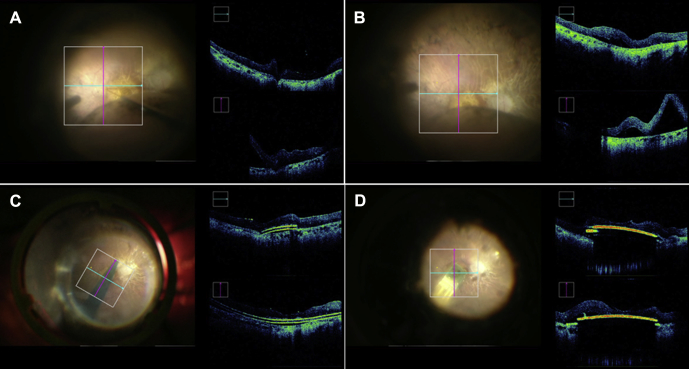
Integrated OCT microscope (Rescan 700, Carl Zeiss Meditec AG, Jena, Germany). The surgeon's perspective through the operating microscope eyepiece (i.e., superior retina is the lower half of the fundus image in each panel) of a left eye. Beside the fundus view is a real-time dual-plane OCT image. Before insertion of the guide foil, a 41-gauge subretinal injection cannula (DORC BV, Zuidland, the Netherlands) was used to induce a superotemporal retinal detachment that extended toward the posterior pole (**A**, **B**). The blue-colored guide foil was advanced under the detached retina (**C**). The retinal implant chip was then positioned under the fovea (**D**).

**Figure 3:**
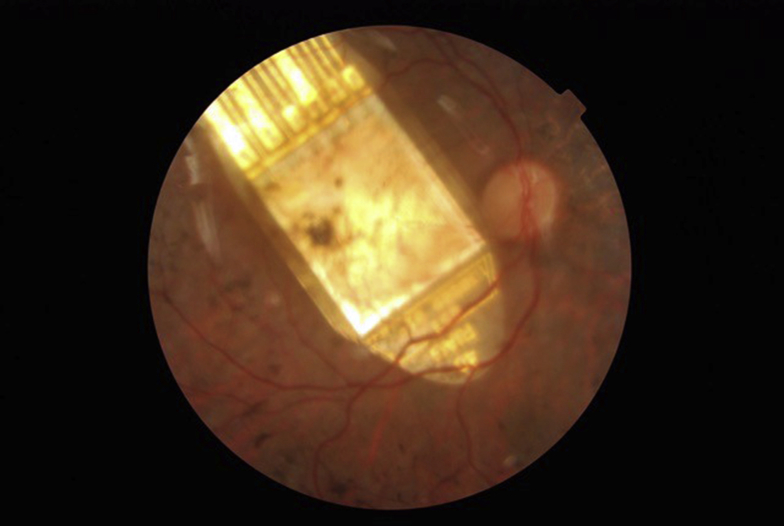
Color fundus photograph of P2 on day 10 postimplantation surgery showing an optimal subretinal chip position centered at the macula.

The first phase of the operation was performed by an experienced cochlear implant surgeon (JDR). A posterior auricular skin and periosteal incision was made, followed by creation of a shallow recess in the mastoid bone into which the ceramic housing of the induction coil was embedded. Next, a skin and periosteal incision over the superolateral orbital rim was made before a trocar was used to create a subcutaneous tunnel between the 2 incisions, enabling passage of the retinal implant and cable from behind the ear to inside the orbit. A 360° conjunctival peritomy followed by blunt dissection to bypass the lacrimal gland was then performed, creating a further passage for the chip and distal cable through orbital fat to the eye. A fornix-based partial-thickness scleral trapdoor was constructed in the superotemporal quadrant (Fig 1) followed by a standard 20-gauge pars plana vitrectomy. A subretinal bleb was created that encompassed the macula and superotemporal retina by injecting balanced salt solution with an extendible 41-gauge subretinal injection cannula (DORC BV, Zuidland, the Netherlands) connected to the viscous fluid injection port of the Constellation Vision System (Alcon, Fort Worth, TX) (Fig 2). The subretinal space was accessed via a linear sclerotomy and choroidotomy at the base of the scleral flap and kept open using Healon (Abbott Medical Optics Inc, Santa Ana, CA). A flexible guide foil was advanced to the subfoveal region, which enabled the retinal implant, embedded within a polyimide foil, to glide over the guide foil to the same location (Fig 1). After positioning the chip array subfoveally (Figs 2 and 3), the guide foil was removed. The connecting cable was sutured to the sclera via an integrated silicone mesh, which was then covered with a scleral patch graft. All eyes received a silicone oil (1300 cSt) endotamponade.

### Clinical End Points

The primary study end point was efficacy of the Alpha AMS when used during activities of daily living and recognition tasks, for example, tabletop tests, clock face recognition, and greyscale discrimination. Secondary end points included objective assessments of light perception, for example, the screen-based Basic Light and Motion (BaLM) test^8^ and detection of gratings of different spatial frequency. Although participants were officially masked as to whether the implant was ON or OFF, flashes evoked by the device typically alerted the participant to the power status. All subjects wore appropriate refractive correction and had the fellow eye covered during testing. Although no strict time limits were imposed on tests, participants were encouraged to provide prompt answers.

### Tabletop Object Recognition

These tests, which have been described,^9^ were designed to replicate visual tasks that might be useful for a severely visually impaired person at home.

*1. Geometric shapes*
  Four white geometric shapes, for example, square, triangle, circle, and oblong (each subtending approximately 5° of visual angle), were positioned on a black tabletop. Participants were required to locate each object and to name the shape; a score of 0 to 4 was recorded for each of these tasks.
*2. Tableware items*
  Four white tableware items simulating a real-life situation, for example, knife, fork, spoon, and plate were similarly positioned on a black tabletop. Both the number of correctly located and named items were recorded. For both tabletop tests, object luminance was 400 to 500 cd/m^2^, background luminance was 30 to 42 cd/m^2^ (Michelson contrast 81%–89%), and illuminance was approximately 2000 lux.

### Clock Face Recognition

Participants were asked to tell the time by correctly identifying the cardinal positions of white clock hands (1.5-cm wide, 5- and 10-cm long) on a 20-cm diameter black analogue clock face laid flat on the tabletop. The examiner presented 12 times in random order.

### Greyscale Contrast

This test^9^ comprised 2 greyscale brightness levels presented side by side on a computer screen, positioned at 60 cm and split in half vertically. Each presentation compared 1 greyscale level on half of the screen (e.g., 0%, 25%, 37.5%, 62.5%, 75%, or 100%) with a 50% greyscale level on the adjacent half. The participant was asked to identify which side was lighter or darker or if they looked equal (i.e., no contrast visible). Each combination was presented 3 times. A 50% greyscale monotone was presented as a negative control on 9 occasions.

### Mobility Questionnaire

A modified Turano Independent Mobility Questionnaire^10^ was used to monitor participants' subjective assessment of their mobility at home in their natural surroundings.

### Basic Light and Motion Test

Light perception, temporal resolution, localization, and motion detection were assessed on a screen at 60 cm after a 2 or 4 alternate forced choice (AFC) algorithm in 8 trials as described previously.^9^ Briefly, the test had 4 elements: (1) identify during which of 2 intervals a single flash of light (200 ms duration) occurred (2-AFC), (2) discern between 1 or 2 flashes (200 ms duration with a 2000 ms interval) of light (2-AFC), (3) localize the quadrant from which a wedge of light was briefly presented (4-AFC), and (4) determine the direction of movement (2.3 degrees/s) of a dot pattern (4-AFC). At least 75% (in 2-AFC) or 62.5% (in 4-AFC) of responses must be correct to pass the test, which resulted in a probability for a false-positive pass of 14.5% (2-AFC) and 2.7% (4-AFC) in an individual test run, based on achieving 6 or more of 8 (2-AFC) or 5 or more of 8 (4-AFC) correct answers. Stimulus luminance was 1400 cd/m^2^.

### Basic Grating Acuity

The Basic Grating Acuity test presented black and white stripes on the viewing screen at 60 cm in a 2-AFC paradigm (e.g., horizontal or vertical). The participant was required to identify the line orientation. Spatial frequency was increased to measure the limits of vision (0.1, 0.3, 1.0, and 3.3 cycles per degree [cpd]). Stimulus luminance was 1400 cd/m^2^.

### Full-Field Stimulus Test

A dark-adapted, achromatic full-field stimulus test (FST) was performed within 3 months of implantation surgery using the Espion ColorDome (Diagnosys, Cambridge, UK). The FST is a psychophysical test of luminance threshold that does not rely on patient fixation and has been used to characterize RP^11^ and macular dystrophies,^12^ being particularly useful when visual fields are poor or electroretinograms nondetectable. The scotopic threshold sensitivity for 50% of responses, determined from the inflection point of the sigmoidal output curve, was compared between implant ON and OFF. To allow for comparable FST responses, the individual chip gain and sensitivity settings that had been optimized during tabletop and screen-based tests were used for each participant. Participants were dark adapted for 45 minutes before commencement of the test.

### Safety Reporting

All adverse events occurring during the study, observed by the investigator or reported by the participant (whether or not due to study medication or implant device) were recorded and if deemed necessary, reported to local authorities and the research ethics committee in accordance with the study protocol. It was mandatory to report any serious adverse event to the research ethics committee, the sponsor (University of Oxford), the Medicines Health Regulatory Agency, and the device manufacturer, Retina Implant AG.

### Statistical Analysis

Because of the small number of participants (n = 6), statistical analysis was limited to calculating mean or median values (and range) for test results with implant ON versus implant OFF. The nonparametric Wilcoxon matched-pairs, signed-rank test was used to determine the statistical significance of implant ON versus implant OFF performance on FST. These data were first transformed on the basis of the percentage of maximum threshold needed to elicit the FST to minimize any ceiling effect and to produce a more meaningful figure.

## Results

The Alpha AMS device was successfully implanted in all 6 participants (Fig 3), 5 of whom achieved the primary end point of improved activities of daily living with the implant switched ON versus OFF, as assessed by recognition of items on a high-contrast tabletop and greyscale discrimination. The same 5 participants reached the secondary end point of improved visual function with the device ON versus OFF using a range of screen-based standardized low vision assessments (BaLM and Basic Grating Acuity). The study protocol prescribed a maximum of 12 months follow-up postimplantation; thus far, this has been accomplished for the first 2 participants (P1 and P2). P5 has reached 6 months of follow-up, and P6 has passed 3 months of follow-up. Gain and sensitivity controls did not function normally at “switch-on” for P4 due to a presumed electrical malfunction of the device. It was subsequently determined that this was the result of microtrauma to the polyimide foil that occurred at the time of primary surgery together with incorrect implantation. In P3, the device stopped working after a secondary conjunctival advancement procedure to cover an area of late-onset foil exposure between months 2 and 3. Subsequent explantation revealed iatrogenic microtrauma to the polyimide foil waterproof seal where it had been refolded to reposition it posteriorly.

### Primary End Points

#### Tabletop Tests

**Figure 4:**
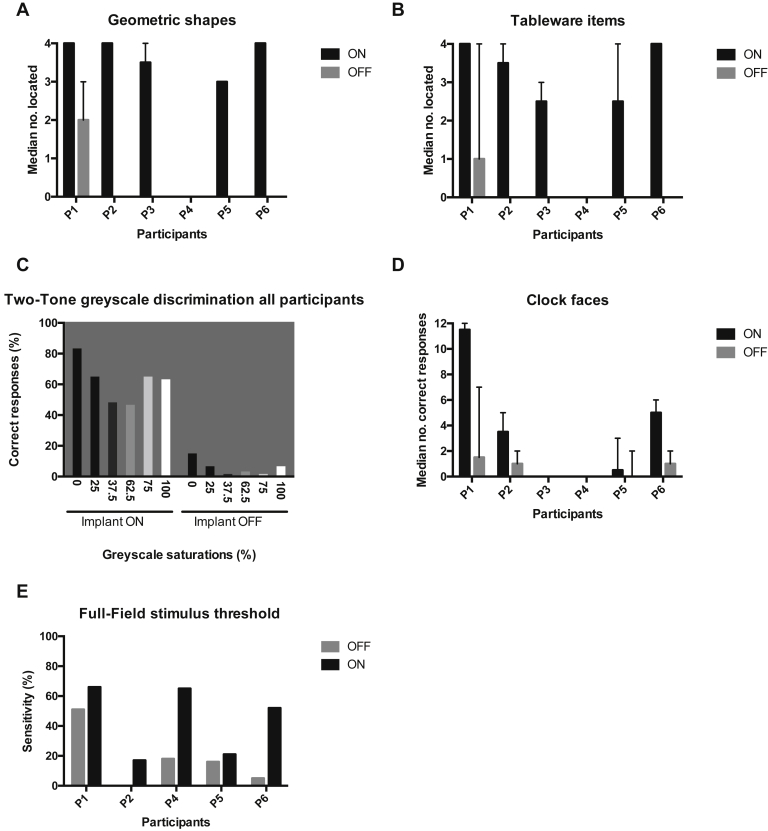
Activities of daily living and recognition tasks. **A**, **B**, The median number of correctly located tabletop items (maximum 4) for all follow-up visits to date, for each participant with implant ON versus OFF. *Absent bars* indicate that nothing was seen. *Error bars* show the range. Greyscale contrast discrimination was explored by testing participants' ability to discern a difference between 1 of 6 greyscale values on one half of a screen—from 0% (*black*) to 100% (*white*)—compared with 50% saturation on the adjacent half of the screen. The percentage of correctly detected contrast pairs is shown (**C**) for all participants except P4, whose implant had malfunctioned. Bars are shaded according to their respective greyscale value and presented on a 50% background to simulate the contrast shown on the screen. Analogue clock face identification was one of the most challenging tests (**D**). **E**, Dark-adapted achromatic full-field stimulus test (FST) (Espion, ColorDome, Diagnosys, Cambridge, UK) using implant gain and sensitivity settings optimized for tabletop testing (i.e., not for scotopic conditions) showed an overall improved threshold sensitivity with the implant ON versus OFF, although it did not reach statistical significance (*P* = 0.06, Wilcoxon matched-pairs, signed-rank test). A reliable threshold with the implant OFF could not be measured for P2. The test was not performed on P3 because of conjunctival erosion.

Five participants (P1, 2, 3, 5, and 6) were able to locate high-contrast objects with the implant ON that they could not localize with the implant OFF. Figure 4A and B show the median number of objects (geometric shapes or tableware; maximum = 4) correctly located on a tabletop for each participant over all postoperative assessment visits to date. By analyzing the 5 chips that were working normally (i.e., excluding P4), the overall mean numbers of geometric and tableware items (maximum 4) located correctly were 3.6 (standard deviation, 0.7) and 3.3 (standard deviation, 0.8), respectively. By month 3, both P1 and P6 were also able to correctly name between 3 and 4 geometric shapes (e.g., square, circle, rectangle, oblong) and tableware items (e.g., knife, plate, spoon, fork) with the implant ON, which neither participant could do with the device switched OFF.

#### Greyscale Contrast Detection

To better understand implant-mediated contrast detection, the percentage of correctly identified greyscale pairs from every test performed in the study, that is, all follow-up visits for all participants with functioning implants (excludes P4), were combined for analysis (Fig 4C). Better performance was observed when the contrast between side-by-side grey values was highest, for example, side-by-side values of 0% and 50%, or 50% and 100%; relatively poorer responses were observed when there was less contrast between these grey values. With the implant ON, the percentage of correct responses for all contrast combinations at all visits for each participant was 61%, 72%, 28%, 65%, and 70% for P1, 2, 3, 5, and 6, respectively. When the implant was OFF, all participants performed poorly: P2, 3, 5, and 6 were unable to discern any contrast combinations at any visits. Consistent with her evident residual native vision, P1 was able to correctly discriminate between side-by-side greyscale levels of 0% and 50% with the implant OFF in 3 of 3 presentations of that combination at months 3, 9, and 12 and had an overall correct response rate of 23% for all contrast combinations with the implant OFF over the 12-month study period. It was not possible to make a reliable comparison between implant ON and OFF for the control screens (which presented just 1 greyscale value). This was because the default answer was “no difference seen,” which led to a high number of false-positive responses to the control screen when the implant was OFF.

#### Clock Face Recognition

This was one of the most challenging tests of the assessment routine. By using cardinal points only (e.g., 9:30, 12:15), 12 different times were presented to participants in random order. Figure 4D shows the results of all follow-up visits for each participant with implant ON versus implant OFF. P1 was the best performer, correctly identifying all 12 presented times at months 3, 6, and 9 compared with 2, 1, and 6 correct times identified at the same visits with the implant OFF. The median number of correctly identified clock faces with the implant ON (and OFF) for the remainder of the cohort were 3.5 (1.0), 0 (0), 0 (0), 0.5 (0), and 5 (1) for P2, 3, 4, 5, and 6, respectively.

### Secondary End Points

#### Basic Light and Motion

**Figure 5:**
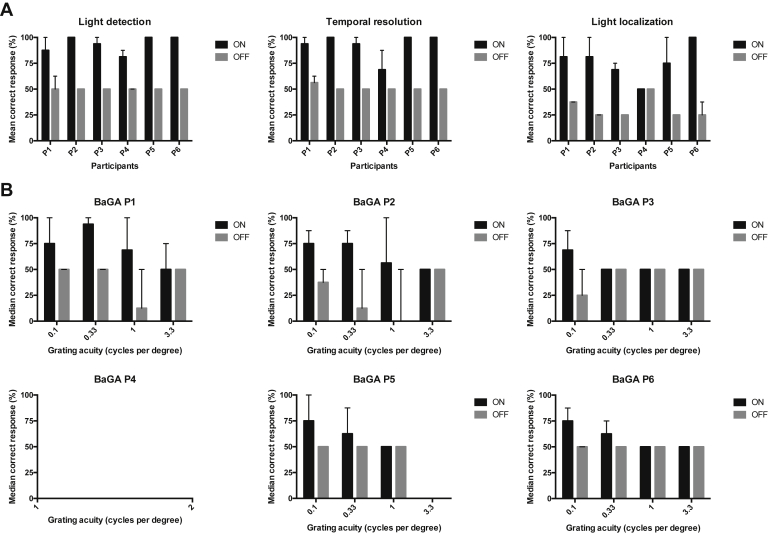
Basic vision assessments. Basic Light and Motion (BaLM) (**A**) and Basic Grating Acuity (BaGA) (**B**) assessments comparing the median response from all visits to date for each participant with implant ON versus OFF. Assessments were conducted as 8 repeated 2 or 4 alternate forced choice (AFC) test (the latter was used in the light localization component of BaLM), requiring a ≥75% or ≥62.5% pass rate. The default result was 50% due to random chance. The probability of a false-positive pass for the 2- and 4-AFC tests was 14% and 2.7%, respectively. No Basic Grating Acuity data were generated from P4 due to chip malfunction. *Error bars* display the range.

All 5 participants with functional chips (i.e., all but P4) passed the light perception (flash or no flash), temporal resolution (1 or 2 flashes of light), and light localization modules of BaLM with the implant ON. None passed the motion component. No participants reached the “pass” threshold with the implant OFF for any element of the test, that is, they tended to perform no better than chance in a 2-AFC test (∼50% correct responses only). Figure 5A shows the median (range) of responses for each participant over all follow-up visits. The performance over all follow-up visits to date for each participant is shown in Figure S1 (available at www.aaojournal.org).

#### Grating Detection

Four spatial frequencies were presented in 2-AFC paradigms: 0.1, 0.33, 1.0, and 3.3 cpd. The highest spatial frequency recorded was 3.3 cpd (P1), although this was achieved only once (6 months postimplantation). At lower spatial frequencies (0.33 and 1.0 cpd), P1 consistently passed the 2-AFC at 6, 9, and 12 months postimplantation (Fig S2, available at www.aaojournal.org). The highest spatial frequencies with orientation correctly detected in the implant ON state for P2 to P6 (and the month it was first recorded) were 1.0 (3 months), 0.1 (2 months), 0 (no response by P4), 0.33 (2 months), and 0.33 cpd (2 months), respectively. No participants did better than chance (i.e., 50% correct response) with the implant OFF. Figure 5B shows the median (range) of correct responses at each spatial frequency for all participants. The performance over all follow-up visits to date for each participant is shown in Figure S2 (available at www.aaojournal.org).

### Additional Tests

#### Full-Field Stimulus Test

To negate fixation instability, an FST was used to assess global chip response between 2 and 3 months postimplantation. The test was not performed in P3 because of her conjunctival revision surgery. Although an FST response was detected in P2 with the implant ON, a reliable OFF FST could not be obtained. A relative improvement in dark-adapted full-field threshold sensitivity was recorded with the implant ON compared with OFF in all participants tested, including P4 (Fig 4E), although the difference did not reach statistical significance (*P* = 0.06, Wilcoxon matched-pairs signed-rank test).

#### Questionnaires

**Figure 6:**
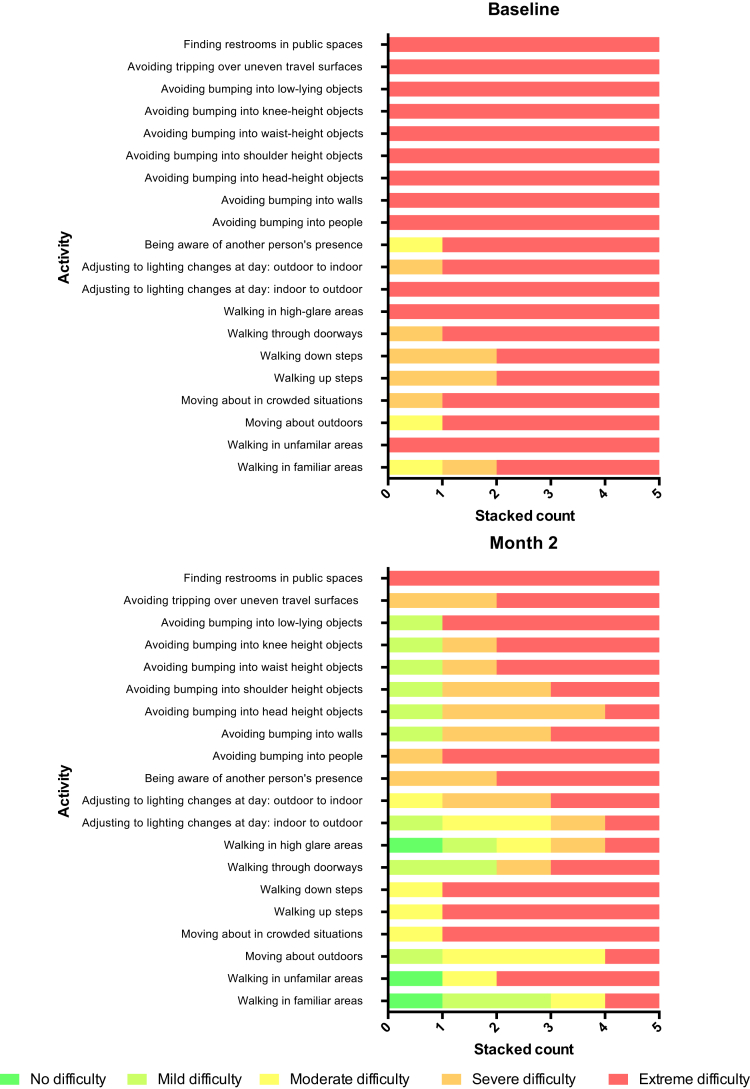
Modified Turano Independent Mobility Questionnaire. The degree of difficulty encountered when performing a range of activities was compared before and 2 months after implantation. “Walking in familiar areas” was the activity that changed most from baseline. The only baseline activity that did not improve was “Finding restrooms in public spaces.” P4 was not included in this analysis because device malfunction noted at switch on severely limited gain and sensitivity controls required to optimize its performance.

To provide an opportunity for participant-reported outcomes, a modified Turano Independent Mobility Questionnaire was performed. Participants were asked to grade the level of difficulty of various activities on a 5-point Likert scale (none, mild, moderate, severe, or extreme difficulty). Figure 6 contrasts participants' baseline self-assessment of mobility with their experience after the first month of implant use (chosen for analysis because it was a time point all study participants had reached at the time of writing). Put into context, none of the 6 participants could mobilize independently outside of their homes, and with respect to mobility aids, P1 used a long cane; P3, P4, and P6 all used guide dogs; and P2 and P5 used neither a cane nor a guide dog. The activity most positively affected by use of the implant was “walking in familiar areas,” with 3 participants noting a more than 3-step improvement on the 5-point difficulty scale. This observation was also persistent, that is, at every follow-up visit, all participants (excluding P4) rated “walking in familiar areas” as less difficult compared with baseline. P6 reported the most dramatic improvement of any participant, with “walking in unfamiliar areas” improving from “extreme difficulty” to “no difficulty” and “walking in familiar areas” improving from “moderate difficulty” to “no difficulty.” Avoidance of bumping into differently sized objects also improved from “extreme difficulty” to “mild difficulty” for P6. At all follow-up visits, overall satisfaction with implant-mediated vision was reported as “sometimes” or “often” better than before surgery by all participants, except P4.

#### Anecdotal Experiences

Participants reported their anecdotal experiences during a range of daily life activities with the implant switched ON during the interval between study visits (Table 2). These included being able to locate or see balls on a billiards table, outlines of windows and doorways, metallic kitchen appliances (e.g., kettle and toaster), black and white laundry items, the outline of a Scottish highland mountain against the setting sun, a passing car, Christmas lights, and building edges. The device was helpful for determining the ambient light level (e.g., whether it was a bright or cloudy day) or when there was shade (e.g., walking under an overhanging tree). An improvement in the vision of the fellow eye was reported by P6 and by P5, who gave a specific example of seeing his hand on 1 occasion using the nonoperated eye.

**Table 2:** Participants' Descriptions of Experience with Implant at Home

Participant	Activities Performed and Objects Seen
P1	Able to find swimming pool
	Tea and coffee machines in restaurant
	Metal-colored kitchen appliances, e.g., kettle, stainless-steel sink
	Able to navigate familiar paths and steps in garden
	Able to see the sea against the sky
	Locate snooker balls on snooker table
	Better in bright conditions
P2	White sockets against black tiles
	Kitchen cupboards
	Steps in home
	Outline of people against backlight
	Plant in sitting room
	Shadows of TV
	Outline of windows
	Can tell when cloudy
	Outlines of cars parked against curb
	Gaps between houses
	Occasionally able to see white line in center of the road
	Black and white socks
P3	Better able to find doorways in home
	Able to tell dark areas, light areas when on walks outside
	Letter on doormat
	Could determine which light switches controlled certain lights in home
	Able to make out shape of horse in field
	Could navigate down laneway without white stick following verge line
	Aware of difference between grass and tarmac
	Friend observed that she had better posture
	Gaps between buildings
P5	Building edges
	Skyline
	Outline of Scottish highlands mountain against setting sun
	Navigate around workshop
	White plates on black background
	Feels that nonoperated eye has improved
P6	Could see Christmas lights
	Window frames
	Car passing on street
	Dog's white harness

#### Safety

**Figure S3:**
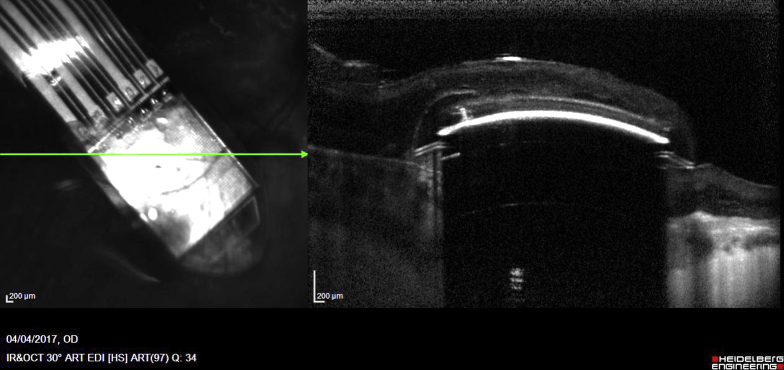
Optical coherence tomography scan (Heidelberg Engineering, Inc., Heidelberg, Germany) of P4 after reimplantation of a new Alpha AMS device (Retina Implant AG, Reutlingen, Germany).

There were 5 adverse events in total, 4 relating to the electronic implant (Table 3). External erosion over the foil was successfully repaired in P1 2 months postimplantation. A similar event occurred twice to P3, and the entire device was subsequently removed, with no adverse sequelae, after inadvertent iatrogenic damage to the laminate insulation of the polyimide foil during conjunctival erosion repair at month 3. A peripheral retinal detachment occurred in the implant eye of P2 at 9 months, but reattachment surgery was performed as a day case without disturbing chip position. A skin rash over the back of P4 developed after her surgery for a contact dermatitis, which resolved within 2 weeks. The implant device in P4 did not function normally from switch at month 1. It was subsequently explanted, and a new (off-study) implant was reinserted at the same operation (Fig S3, available at www.aaojournal.org). Subjective (off-study) descriptions of the new device in use included indications of improved performance, for example, seeing her hands, including her wedding ring, against the black coat of her guide dog. In the evenings, P4 reported identifying streetlights, shop-front windows, and lights through her house window as she approached the front door. On 1 occasion she was able to recognize a sunset.

**Table 3:** Adverse Events

Participant	Adverse Event	Time of Onset Postimplantation
P1	Conjunctival erosion	Mo 2
P2	Retinal detachment	Mo 9
P3	Conjunctival erosion	Mo 3
P4	Skin rash	Day 1 (resolved within 2 wks)
	Device failure (incorrect implantation procedure)	Noted at mo 1

## Discussion

The Alpha AMS subretinal implant provided objective and participant-reported functional benefit to this NHS-based patient cohort with profound blindness secondary to RP. As with the antecedent Alpha IMS retinal implant device,^9^ preliminary assessment of the AMS implant in this Oxford-based study has shown that the electronic subretinal prosthesis can provide measurable visual gains in a specific group of patients with RP, that is, those with residual inner retinal function. Improvements in vision-related quality of life and real-world function have been reported in larger cohorts with the Argus II epiretinal prosthesis.^13^, ^14^ Although no head-to-head trial between the 2 devices has been conducted, introduction of standardized assessment protocols for extreme low vision^15^, ^16^ may facilitate future comparison between different retinal prostheses. There are presently no data available on clinical trial outcomes for the IRIS II device.

A relatively intact inner retina is required to transmit light-induced chip activity to the visual pathway. The utility of both external or internal electrical retinal stimulation to predict the sensitivity of the human visual system has been reported in a number of disease states.^17^, ^18^ Because a degree of ganglion cell loss undoubtedly occurs in end-stage RP,^19^ it was critical to screen potential participants for residual inner retinal function with an electrically evoked phosphene test; absent electrically evoked phosphene tests were a study exclusion criterion.

Thus far, the Alpha AMS has exhibited superior longevity compared with its predecessor, the Alpha IMS, in the present study and in laboratory tests.^20^ Of note, the reasons for failure were iatrogenic damage during revision surgery in P3 and damage to the foil and incorrect insertion technique in P4, rather than erosion of electrical insulation as was seen in the Alpha IMS, which bodes well for future clinical applications. Although IMS results were significantly better with the implant ON compared with the implant OFF in the first 3 months, a number of the former devices failed after 3 to 12 months.^9^ By comparison, the Alpha AMS has been functioning now for more than 24 months in the first 2 participants recruited to the trial (P1 and P2).

In P4, the implant gain and sensitivity controls did not respond at device “switch on,” severely limiting its utility for testing and general-purpose use between follow-up visits (some limited function was detected on the BaLM and FST tests only). A decision was made to explant the subretinal device as an “off-study” procedure after 9 months. This was the first time a same eye Alpha AMS combined explantation and implantation procedure had been performed. Reassuringly, the new device has since functioned normally, providing subjective visual benefits to daily activities. Iatrogenic damage to the polyimide foil protective coating was responsible for device failure in P3; however, before this, the device had been functioning well, for example, allowing P3 to identify her horse in a field and to discern a white envelope on the front doormat. She was also able to navigate independently down a country lane with the chip switched ON. The likely cause of conjunctival retraction and foil exposure was tension at the limbal wound due to anterior prominence of the scleral patch. Although alternatives to scleral allograft exist (e.g., temporalis fascia autograft^21^), the risk of wound dehiscence was ameliorated in subsequent cases by construction of a 2-mm corneoscleral pocket to receive the anterior most edge of the scleral graft, thus smoothing the conjunctival profile and lessening tissue tension in the vicinity of the limbal conjunctival wound. It is likely that surgical techniques will improve with further refinements as we gain more experience with retinal implant surgery.

Estimating maximum theoretic chip-mediated visual acuity is based on stimulation unit density and chip dimensions. A spatial resolution of 16 minutes of arc (0.26 degrees) and a visual field of approximately 15 degrees (diagonally) are potentially obtainable with the Alpha AMS chip. A participant using an Alpha AMS implant could theoretically gain a best-corrected visual acuity of approximately 6/96, but this was not achieved by any participants in this study. The best performance on detecting grating orientation testing was from P1, who could discern the directions of gratings with a spatial frequency of 3.33 cpd, a theoretic logarithm of the minimum angle of resolution (logMAR) equivalent of 0.95 and approximate Snellen acuity of 6/60. However, the patient was unable to reliably discern Landolt C letters on further testing, suggesting a disparity between the 2 methods of acuity determination; furthermore, she achieved this result on only 1 occasion (at 6 months). Two-point discrimination and optotype recognition ability for very low vision cannot directly be compared with grating acuity because the detection of long lines involves multiple—possibly even dispersed—photosensor areas, the elongated stimulation of which can be recognized as lines. All participants with functioning implants achieved at least a logMAR equivalent of 2.48 (0.1 cpd), which compared favorably with a cohort of Argus II patients in whom 48% scored a grating acuity–derived logMAR of 2.9 or better at 1 year.^22^ The best reported comparable acuity attained by the Argus II system was 1.6 logMAR, or 20/1262 (6/379).^23^ The best grating acuity reported from the Alpha IMS was 3.3 cpd (0.96 logMAR) in 1 patient who reached 20/546 (6/164) Snellen equivalent (1.4 logMAR) on Landolt C-ring testing.^9^ However, it is important to note that the Argus II trial used a 4-AFC test, rather than the 2-AFC test used here, which was limited to 5 seconds. Furthermore, the psychophysical staircase criterion used to adjust the grating width in the Argus II test was extremely strict, for example, subjects whose grating acuity was reported as >2.9 logMAR achieved that result because they could not maintain a threshold below 2.7 logMAR for up to 40 trials.

Localized iatrogenic detachment of degenerate retina has been performed for the subretinal delivery of adeno-associated viral gene therapy vectors for *RPE65* and *MERTK*-associated RP,^24^, ^25^, ^26^, ^27^ and choroideremia.^28^ In this study, a significantly larger retinal detachment was required to permit safe passage of the AMS chip and PI foil toward the posterior pole, passing underneath pigmented degenerate retina. The benefit of using an integrated OCT surgical microscope for implantation of the epiretinal Argus II Retinal Prosthesis System was recently reported.^29^ It was similarly effective in facilitating submacular chip implantation with enhanced accuracy and surgeon confidence in our study. Specifically, it was most helpful when initiating the retinal detachment before implant insertion and for visualizing safe advancement of the guide foil or chip in the correct tissue plane, between the retina and retinal pigment epithelium. Our positive experience with this technology will see its use mandated in all future similar cases at this center.

In summary, this preliminary report has shown that the recently CE-marked Alpha AMS retina implant performed at least as well as the previous generation Alpha IMS device in a small cohort of patients with end-stage RP. Critically, the new model has thus far demonstrated superior longevity to the IMS device. Advances in ophthalmic technology (e.g., use of the integrated OCT operating microscope) lessened the difficulty of the most technically demanding step of the procedure—subretinal positioning of the chip—making the surgery relatively safer and more predictable.

## Supplementary Data

Figure S1 Change in Basic Light and Motion (BaLM) test over time. The percentage of correct responses recorded for implant ON and OFF at each attended follow-up visit (1, 2, 3, 6, 9, and 12 months) for all participants to date.

Figure S2 Change in Basic Grating Acuity test over time. The percentage of correct responses recorded for implant ON and OFF at each attended follow-up visit (1, 2, 3, 6, 9, and 12 months) for all participants to date. The *horizontal dashed line* denotes the default 50% correct response rate due to chance alone.

## References

[1] Liew G., Michaelides M., Bunce C. (2014). A comparison of the causes of blindness certifications in England and Wales in working age adults (16-64 years), 1999-2000 with 2009-2010. BMJ Open.

[2] Zrenner E. (2013). Fighting blindness with microelectronics. Sci Transl Med.

[3] Schatz A., Pach J., Gosheva M. (2017). Transcorneal electrical stimulation for patients with retinitis pigmentosa: a prospective, randomized, sham-controlled follow-up study over 1 year. Invest Ophthalmol Vis Sci.

[4] Rothermel A., Liu L., Aryan N.P. (2017). A CMOS chip with active pixel array and specific test features for subretinal implantation. IEEE J Solid-State Circuits.

[5] Zrenner E. (2002). Will retinal implants restore vision?. Science.

[6] Zrenner E., Bartz-Schmidt K.U., Benav H. (2011). Subretinal electronic chips allow blind patients to read letters and combine them to words. Proc Biol Sci.

[7] Stingl K., Bartz-Schmidt K.U., Besch D. (2013). Artificial vision with wirelessly powered subretinal electronic implant alpha-IMS. Proc Biol Sci.

[8] Bach M., Wilke M., Wilhelm B. (2010). Basic quantitative assessment of visual performance in patients with very low vision. Invest Ophthalmol Vis Sci.

[9] Stingl K., Bartz-Schmidt K.U., Besch D. (2015). Subretinal visual implant Alpha IMS—clinical trial interim report. Vision Res.

[10] Turano K.A., Geruschat D.R., Stahl J.W., Massof R.W. (1999). Perceived visual ability for independent mobility in persons with retinitis pigmentosa. Invest Ophthalmol Vis Sci.

[11] Messias K., Jagle H., Saran R. (2013). Psychophysically determined full-field stimulus thresholds (FST) in retinitis pigmentosa: relationships with electroretinography and visual field outcomes. Doc Ophthalmol.

[12] Collison F.T., Fishman G.A., McAnany J.J. (2014). Psychophysical measurement of rod and cone thresholds in Stargardt disease with full-field stimuli. Retina.

[13] Duncan J.L., Richards T.P., Arditi A. (2017). Improvements in vision-related quality of life in blind patients implanted with the Argus II Epiretinal Prosthesis. Clin Exp Optom.

[14] Dagnelie G., Christopher P., Arditi A. (2016). Performance of real-world functional vision tasks by blind subjects improves after implantation with the Argus® II retinal prosthesis system. Clin Exp Ophthalmol.

[15] Finger R.P., McSweeney S.C., Deverell L. (2014). Developing an instrumental activities of daily living tool as part of the low vision assessment of daily activities protocol. Invest Ophthalmol Vis Sci.

[16] Finger R.P., Tellis B., Crewe J. (2014). Developing the Impact of Vision Impairment-Very Low Vision (IVI-VLV) questionnaire as part of the LoVADA protocol. Invest Ophthalmol Vis Sci.

[17] Horsager A., Greenwald S.H., Weiland J.D. (2009). Predicting visual sensitivity in retinal prosthesis patients. Invest Ophthalmol Vis Sci.

[18] Gekeler F., Messias A., Ottinger M. (2006). Phosphenes electrically evoked with DTL electrodes: a study in patients with retinitis pigmentosa, glaucoma, and homonymous visual field loss and normal subjects. Invest Ophthalmol Vis Sci.

[19] Jones B.W., Pfeiffer R.L., Ferrell W.D. (2016). Retinal remodeling in human retinitis pigmentosa. Exp Eye Res.

[20] Daschner R., Greppmaier U., Kokelmann M. (2017). Laboratory and clinical reliability of conformally coated subretinal implants. Biomed Microdevices.

[21] Matet A., Amar N., Mohand-Said S. (2016). Argus II retinal prosthesis implantation with scleral flap and autogenous temporalis fascia as alternative patch graft material: a 4-year follow-up. Clin Ophthalmol.

[22] da Cruz L., Dorn J.D., Humayun M.S. (2016). Five-year safety and performance results from the Argus II Retinal Prosthesis System Clinical Trial. Ophthalmology.

[23] Humayun M.S., Dorn J.D., da Cruz L. (2012). Interim results from the international trial of Second Sight's visual prosthesis. Ophthalmology.

[24] Hauswirth W.W., Aleman T.S., Kaushal S. (2008). Treatment of Leber congenital amaurosis due to *RPE65* mutations by ocular subretinal injection of adeno-associated virus gene vector: short-term results of a phase I trial. Hum Gene Ther.

[25] Maguire A.M., Simonelli F., Pierce E.A. (2008). Safety and efficacy of gene transfer for Leber's congenital amaurosis. N Engl J Med.

[26] Ghazi N.G., Abboud E.B., Nowilaty S.R. (2016). Treatment of retinitis pigmentosa due to *MERTK* mutations by ocular subretinal injection of adeno-associated virus gene vector: results of a phase I trial. Hum Genet.

[27] Bainbridge J.W.B., Smith A.J., Barker S.S. (2008). Effect of gene therapy on visual function in Leber's congenital amaurosis. N Engl J Med.

[28] Edwards T.L., Jolly J.K., Groppe M. (2016). Visual acuity after retinal gene therapy for choroideremia. N Engl J Med.

[29] Rachitskaya A.V., Yuan A., Marino M.J. (2016). Intraoperative OCT imaging of the Argus II Retinal Prosthesis System. Ophthalmic Surg Lasers Imaging Retina.

