# Supplementary figures and images for: Assessment of the Electronic Retinal Implant Alpha AMS in Restoring Vision to Blind Patients with End-Stage Retinitis Pigmentosa
Source: Ophthalmology. 2018 Mar;125(3):432–43. doi: 10.1016/j.ophtha.2017.09.019 (PMC5818267; doi:10.1016/j.ophtha.2017.09.019)

A) Implant ON

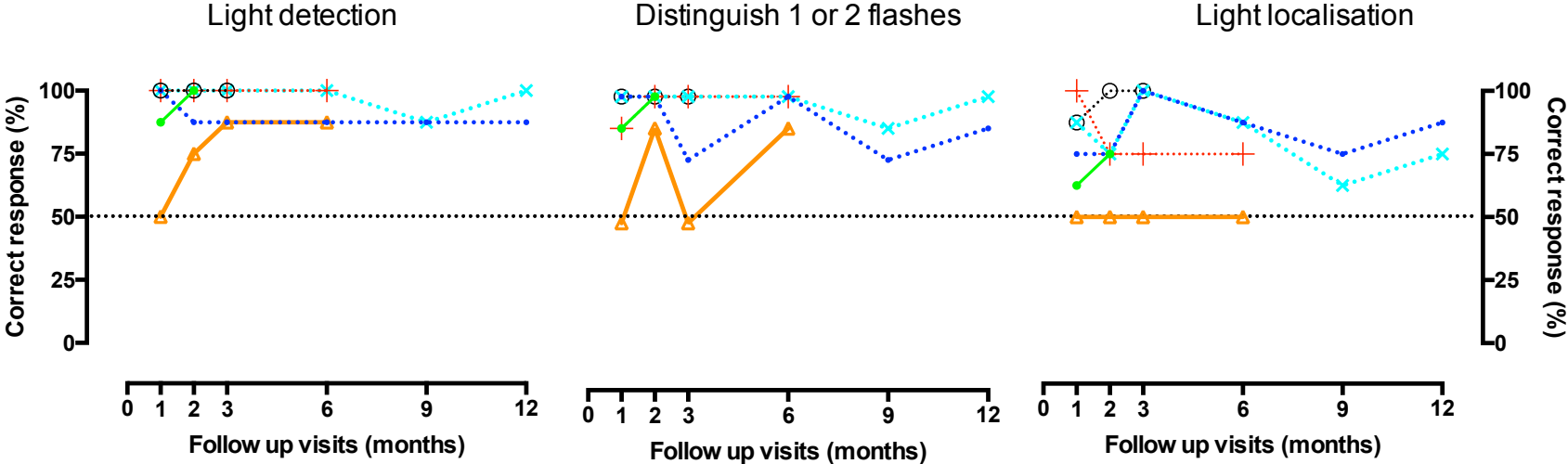

B) Implant OFF

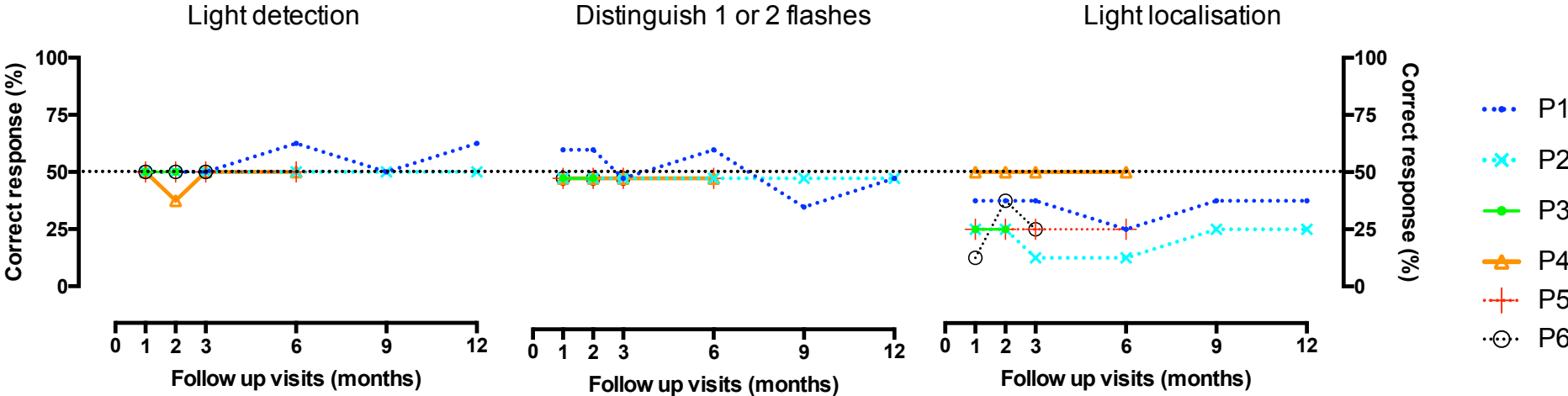

Supplement: Figure S1 — Change in Basic Light and Motion (BaLM) test over time. The percentage of correct responses recorded for implant ON and OFF at each attended follow-up visit (1, 2, 3, 6, 9, and 12 months) for all participants to date. [file mmc1.pdf]

## A) Implant ON

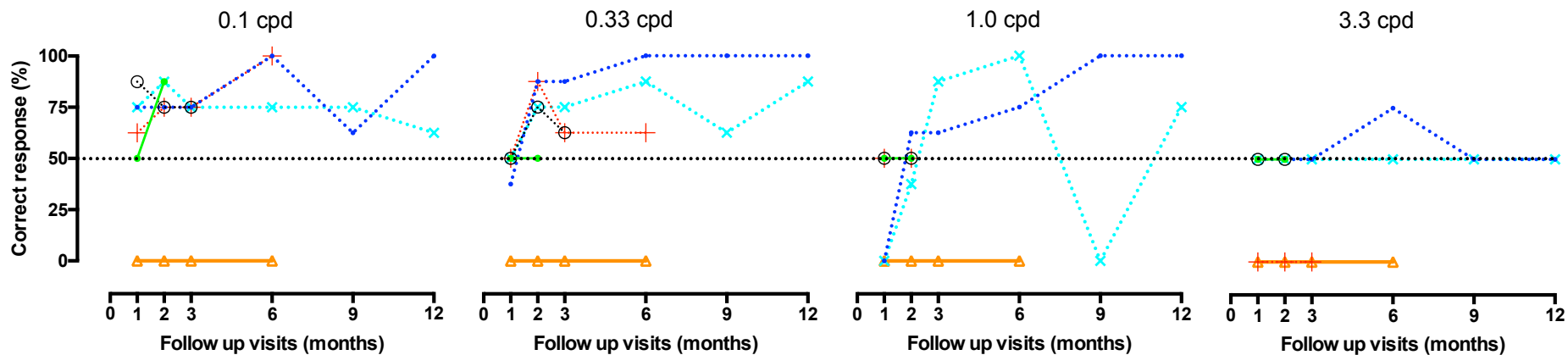

## B) Implant OFF

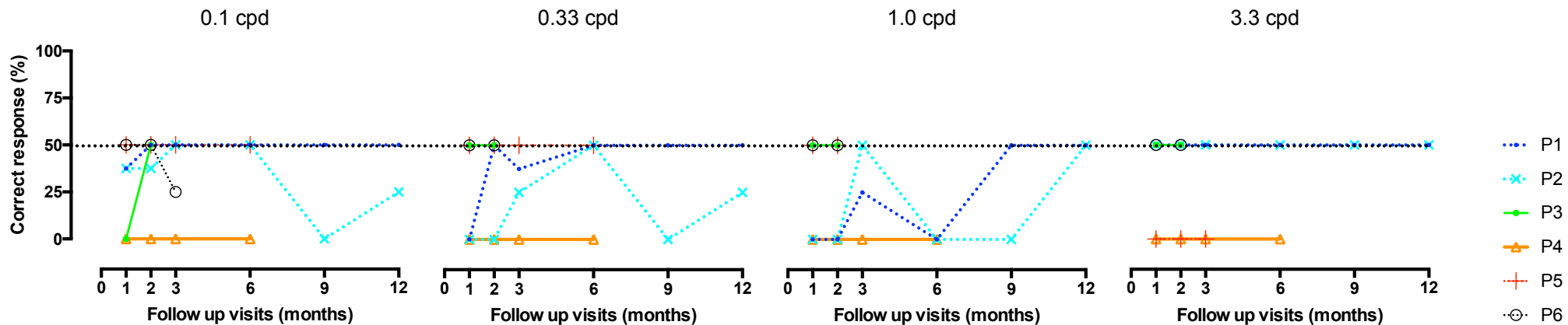

Supplement: Figure S2 — Change in Basic Grating Acuity test over time. The percentage of correct responses recorded for implant ON and OFF at each attended follow-up visit (1, 2, 3, 6, 9, and 12 months) for all participants to date. The horizontal dashed line denotes the default 50% correct response rate due to chance alone. [file mmc2.pdf]
